# An Innovative Material with Strong Frost Resistance—Concrete Containing Dolomite Powder

**DOI:** 10.3390/ma15051721

**Published:** 2022-02-25

**Authors:** Xin Zhang, Yu Luo, Wu Yao

**Affiliations:** 1 Key Laboratory of Advanced Civil Engineering Materials, Tongji University, Ministry of Education, Shanghai 201804, China; 1810352@tongji.edu.cn; 2 Qianxinan Urban Construction Investment Co., Ltd., Guizhou 562400, China; luodaguai@163.com

**Keywords:** concretes, dolomite powder, pore structure, freeze–thaw cycle, Weibull distribution

## Abstract

The effects of dolomite powder on the freeze–thaw resistance of C30 and C45 concrete were investigated in this manuscript. Scanning electron microscopy (SEM), the electric flux method, and a freeze–thaw cycle testing machine were used to determine the morphology, chloride penetration resistance, mass loss rate, and relative dynamic elastic modulus ($E_{r}$) of concrete incorporating dolomite powder. Concrete’s freeze–thaw resistance improved as the dosage of dolomite powder was increased. After 300 cycles, the mass loss rates of reference specimens of C30 and C45 concrete were 6.71% and 0.14%, respectively, whereas the mass loss rates of C30 and C45 concrete in the presence of dolomite powder at a 50% replacement level were 5.81% and 0.13%, respectively. After 225 cycles, the $E_{r}$ of C30 concrete was 42.57% and 48.56% in the case of dolomite powder at 0 and 50% replacement levels, respectively. Meanwhile, after 300 cycles, the $E_{r}$ of C45 concrete was 67.54% and 71.50% in the case of dolomite powder at 0 and 50% replacement levels, respectively. Accordingly, the structure of dolomite-containing cement-based materials became more compact. Based on the Weibull distribution, a damage model for concrete containing dolomite powder was proposed. It established that concrete treated with dolomite powder had a lower degree of damage than reference specimens when subjected to the same freeze–thaw conditions.

## 1. Introduction

Dolomite is an essential carbonate rock, a compound composed of calcium, magnesium, and carbonate ions with the formula CaMg(CO_3_)_2_ that can act as a source of carbon dioxide and magnesium [1,2,3]. Dolomite was discovered to be widely distributed throughout the world, with over 4 billion tons discovered in China [4,5]. Previously, dolomite was considered an inert material and was calcined at high temperatures to be used as a building material. Magnesium cement was made by combining dolomite and magnesite that had been calcined [6]. Xie et al. discovered that cementitious materials containing light-burned dolomite had a higher strength than cement blended with dolomite [7]. Numerous correlations between aggregate minerals and concrete specimens have been reported by some researchers [8,9]. When dolomite was used as coarse aggregate in concrete, it was demonstrated that an alkali–aggregate reaction could occur [10]. Thus, some researchers ground dolomite into powder and then added it as a mineral admixture to cement-based materials [11]. The substitution of dolomite powder for cement clinker facilitated energy savings and environmental protection.

Recently, more attention has been paid to the research of dolomite powder as a mineral admixture for cement-based materials. Numerous studies established that dolomite powder influenced the working properties, cement hydration process, and mechanical properties of concrete [12,13]. Yan et al. discovered that as the specific surface area of dolomite powder increased, the fluidity and dry shrinkage of mortar increased as well [14]. Gali et al. and Zhang et al. [15,16] reported that the rate of dolomite powder dissolution determined the rate of the dedolomitization reaction and that the dolomite dissolution and dedolomitization reactions could be accelerated at a higher temperature. Tian et al. investigated the effect of dolomite powder fineness on the hydration properties of cement paste. The results indicated that increasing the fineness of dolomite powder could result in a decrease in the amount of calcium hydroxide due to an increase in the reaction degree [17]. The hydration process was investigated by Krishnan et al. in a Portland cement-calcined clay-dolomite system. At 3 and 7 days, dolomite calcined clay cement demonstrated greater compressive strength than quartz calcined clay cement [18]. After 90 days of curing at 40 and 60 °C, Xu et al. discovered that mortars containing dolomite powder had a higher compressive strength than mortars containing limestone powder. Accordingly, hydrotalcite and calcite formation aided in the refinement of pore structure [19]. However, there are relatively few studies on the durability of dolomite-powder concrete. Enhancing the durability of concrete was a significant scientific challenge [20,21]. Thus, it is necessary to investigate the durability of dolomite-powder-containing concrete in future work, including resistance to chloride ion permeability, freeze–thaw resistance, sulfate resistance, and carbonation resistance.

Freeze–thaw resistance is a critical indicator of concrete’s durability. In cold regions, freeze–thaw damage to concrete is common, resulting in building destruction and collapse [22]. Hydraulic and osmotic pressures cause cement-based materials to expand during a freeze–thaw cycle. Disruptive pressures result in the formation of cracks and microcracks as the water inside the capillary pore freezes [23]. The freeze–thaw cycle deterioration of concrete structures is a common occurrence, and it has a direct effect on the working performance and service life of concrete [24]. Thus, it is necessary to investigate the life prediction of concrete by simulating the freeze–thaw cycle test. However, there are only a few papers that discuss the evolution model of the damage degree of concrete incorporating dolomite powder during the freeze–thaw cycle.

The Weibull distribution had an increasing hazard function with time and was frequently used to describe the fatigue data. The study showed that the Weibull distribution was adequate to describe the failure behavior of concrete [25,26]. One service life prediction model for the concrete based on the Weibull distribution was established in single freeze-thaw as well as sulfate solution and freeze-thaw environments [27]. The indoor accelerated life test was designed to simulate saline soil area by Qiao et al [28]. The distribution overview plot could reflect accelerated degradation process of concretes according to three-parameter Weibull Distribution. Zhou et al and Anush et al discussed two-parameter Weibull probability distribution model through data analysis. It was found that this model was suitable and the fatigue damage characteristics of pervious concretes were analyzed [29,30]. Thus, the life prediction function of Weibull distribution was widely used in various fields. Based on two-parameter Weibull distribution, the relationship between damage degree and the number of freeze-thaw cycle in this work was established to provide references for the evolution model of freeze-thaw damage and life prediction of concretes incorporating dolomite powder.

The purpose of this manuscript is to examine the freeze–thaw resistance properties of C30 and C45 concrete in which 0, 30 wt%, and 50 wt% of mortar was replaced with dolomite powder. Based on a two-parameter Weibull probability function, an evolution model for the damage degree of concrete incorporating dolomite powder was established. The primary objective was to predict and evaluate the service life of concrete blended with dolomite powder using an evolution model of the degree of concrete damage caused by freeze–thaw cycles. The SEM technique, electric flux method, and freeze–thaw cycle testing machine were used to determine the product morphology of cement-based materials, chloride penetration resistance, mass loss rate, and relative dynamic elastic modulus of concrete.

## 2. Experimental Details

### 2.1. Materials and Mixture Preparation

**Figure 1:**
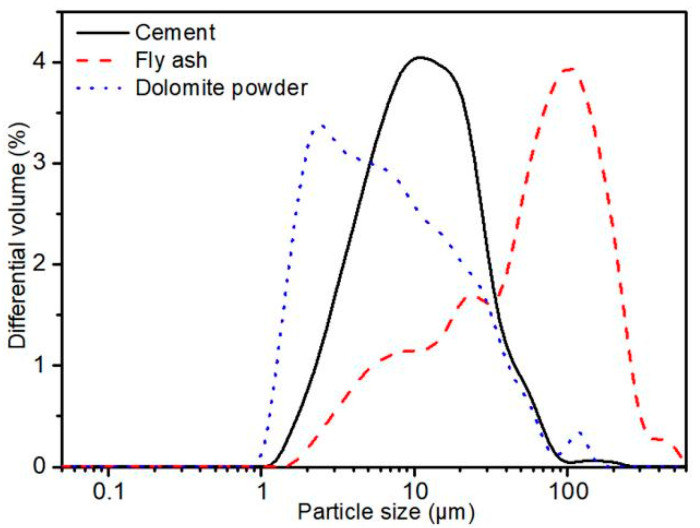
The particle size distribution of cement, fly ash and dolomite powder by the laser diffractometry.

Portland cement (P I 42.5), dolomite powder and fly ash were used in this manuscript, and their specific densities were 3.10, 2.88, 2.60 g/cm^3^, respectively. The dolomite was natural rock supplied by Guizhou Province, China. Coarse aggregate used in this work was crushed basalt from Huzhou xinkaiyuan gravel Co., Ltd. The chemical compositions of Portland cement, dolomite powder and fly ash characterized by means of XRF(Zetium, Malvern Panalytical B. V., Almelo, The Netherlands) were given in Table 1, Table 2 and Table 3, respectively. The mix proportions and identifications of cement pastes and concretes with three dolomite powder contents (0, 30 wt%, 50 wt%) were seen in Table 4 and Table 5. In this work, PDF0, CD0-L and CD0-H were reference specimens. The particle size distribution of cement, dolomite powder and fly ash was characterized by laser diffraction (LS230, Beckman Coulter, Brea, CA, USA). The specimens were measured between 40 nm and 2000 μm. The values of median particle size (D50) for Portland cement, dolomite powder and fly ash were 11.37, 6.45 and 2.88 μm, respectively (see Figure 1).

The cement pastes were made for SEM measurement (TM4000, Hitachi Limited, Tokyo, Japan). C30 and C45 concretes were cast for the mass loss rate and relative dynamic elastic modulus tests under the freeze–thaw cycles. The water-to-binder ratio in cement paste was 0.36. It was noted that binder materials in this work included cement and fly ash. C30 and C45 concretes with two water to binder ratios (0.55 and 0.36) were prepared. Superplasticizer was used to maintain a stable fluidity due to the high fineness of dolomite powder.

### 2.2. SEM Test

To examine the microstructure of the specimen, the accelerating voltage was set as 15 KV. Slices of the hydrated cement paste were cut. The immersion time of the slices in ethanol absolute lasted for one week. Subsequently, the slices were dried overnight at 40 °C and then stored in a vacuum desiccator. Eventually, a piece of slice was examined with the SEM (TM4000, Hitachi Limited, Tokyo, Japan).

### 2.3. Test Design of Chloride-Penetration Resistance

The chloride-penetration resistance of concretes mixed with dolomite powder was performed using the electric flux method. Specimens of cylinders with a diameter of 100 mm were cast. The specimens were taken out from the standard curing room after 28 days of curing, then the specimens were saturated with water in vacuum before the measurement. The measurement was repeated at three times for reproducibility by concrete electric flux meter (TR-SDL, Shanghai Tongrui Instrument Equipment Co., Ltd., Shanghai, China). The electric flux value of each specimen was calculated according to GB/T50082-2009 [31].

### 2.4. Test Design of Frost Resistance Durability

Concretes were made according to a quick freezing method following the standards (GB/T50082-2009) [31]. Three prism specimens of 400 × 100 × 100 mm^3^ were cast. The measurement was repeated at three times for reproducibility by concrete rapid freezing and thawing testing machine (TR-TDRF-28, Shanghai Tongrui Instrument Equipment Co., Ltd., Shanghai, China). The specimens were taken out from the standard curing room after 24 days of curing, and then the specimens were immersed in water for 4 days, i.e., the freeze–thaw test of the specimens was performed after 28 days of curing. The temperature of the specimen center ranged from (−18 ± 2) °C to (5 ± 2) °C. During every 25 cycles, the damage shapes, mass loss rate and relative dynamic elastic modulus of concretes with different compositions under the freeze–thaw cycles were tested.

### 2.5. Mass Loss Rate Test

The mass loss rate test was performed in accordance with GB/T50082-2009 [31]. Before placing the specimens into the freeze–thaw test box, the initial mass of the specimens was weighed. During every 25 cycles, the mass of the specimens was measured. The mass loss rate was calculated using the Equations (1) and (2) as given below:

(1) $$\Delta W_{ni}=\frac{W_{oi}-W_{ni}}{W_{oi}}\;\times \;100$$

In which

$\Delta W_{ni}$: Mass loss rate of specimen *i* after *n* freeze–thaw cycles, %;

$W_{oi}$: Mass of specimen *i* before the freeze–thaw test, g;

$W_{ni}$: Mass of specimen *i* after *n* freeze–thaw cycles, g.

(2) $$\Delta W_{n}=\frac{\sum_{i=1}^{3}\Delta W_{ni}}{3}\;\times \;100\;$$

In which

$\Delta W_{n}$: Average mass loss rate of a group of specimens (three parallel specimens) after n freeze–thaw cycles, %.

### 2.6. Relative Dynamic Elastic Modulus Test

The relative dynamic elastic modulus test was performed based on GB/T50082-2009 [31]. The size of specimens was 400 × 100 × 100 mm^3^. During every 25 cycles, the relative dynamic elastic modulus of the specimens was measured by dynamic elastic modulus testing machine (DT-20, Jinan Langrui Testing Technology Co., Ltd., Jinan, China). The relative dynamic elastic modulus was calculated using the Equation (3) as given below:

(3) $$E_{i}=\frac{f_{ni}^{2}}{f_{n0}^{2}}\;\times \;100$$

In which

$E_{i}$: The relative dynamic elastic modulus of specimen *i* after *n* freeze–thaw cycles, %;

$f_{ni}$: Transverse fundamental frequency of specimen *i* after *n* freeze–thaw cycles, Hz;

$f_{n0}$: Transverse fundamental frequency of specimen *i* before the freeze–thaw test, Hz.

### 2.7. Establishment of Damage Model of Concretes Incorporating Dolomite Powder Based on Two-Parameter Weibull Distribution

The freeze–thaw damage of the concretes resulted in declines of the relative dynamic elastic modulus, which reflected the structure changes inside the concrete. Thus, the $E_{r}$ was selected to describe the degradation rules of concretes blended with dolomite powder. Combined with damage mechanics, the degree of the freeze–thaw damage could be expressed as [32]:

(4) $$D_{N}=1-E_{i}$$

In which

$D_{N}$: The freeze–thaw damage degree;

$E_{i}$: The relative dynamic elastic modulus of specimen i after n freeze–thaw cycles.

The Weibull distribution had an increasing hazard function with time and was commonly used for describing the process of material failure [33]. In this manuscript, the Weibull distribution was used to study the damage model of concretes incorporating dolomite powder. The equation of two-parameter Weibull distribution was defined as:

(5) $$F_{N}=1-exp[-\left(\frac{N}{\alpha }{)}^{\beta }\right]$$

In which

$F_{N}$: The distribution function of the two-parameter Weibull probability model;

N: The number of the freeze–thaw cycle;

α: The scale parameter;

β: The shape parameter.

When the Weibull distribution was suitable to describe the service life of concretes incorporating dolomite powder under the freeze–thaw cycles, the freeze–thaw damage degree was equivalent to the distribution function. The equation was expressed as:

(6) $$D_{N}=F_{N}=1-exp[-\left(\frac{N}{\alpha }{)}^{\beta }\right]$$

Taking the logarithm twice of both sides of Equation (6):

(7) $$\beta \mathrm{In}N-\beta \mathrm{In}\alpha =\mathrm{In}\left(\mathrm{In}\frac{1}{1-D_{N}}\right)$$

To simplify the calculation, mathematical transformation was made:

(8) $$Y=\mathrm{In}\left(\mathrm{In}\frac{1}{1-D_{N}}\right),\;X=\mathrm{In}N,\;a=-\beta \mathrm{In}\alpha$$

Equation (8) could be written in the following form:

(9) Y= βX+a

## 3. Results and Discussion

### 3.1. SEM Test

**Figure 2:**
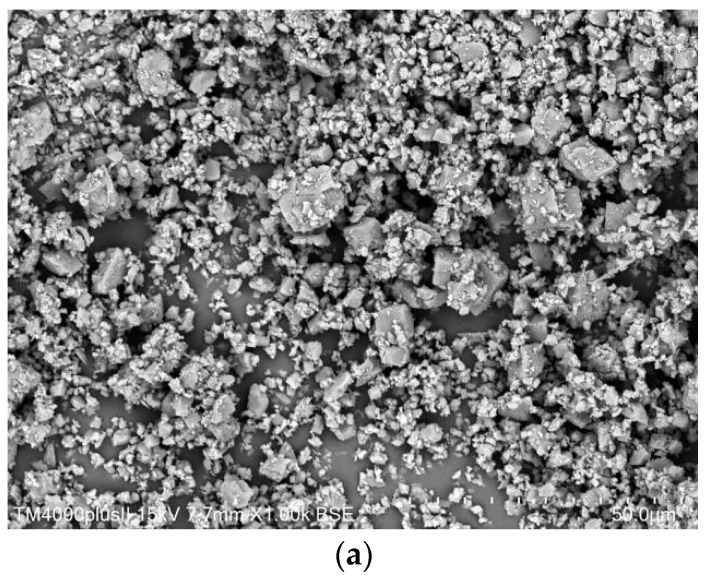

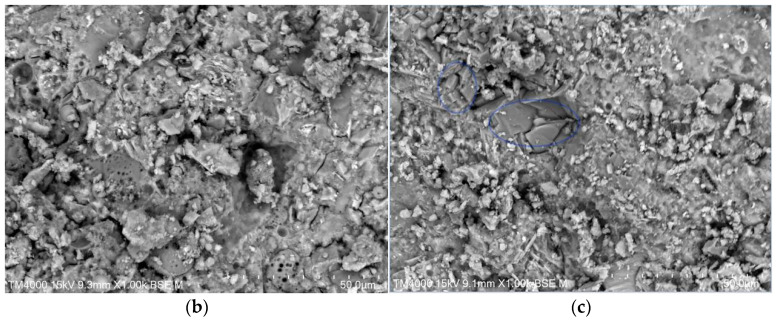
The morphology of specimens: (**a**) dolomite powder; (**b**) PDF0 at 90 days; (**c**) PDF5 at 90 days.

Figure 2a depicts the morphology of dolomite powder. The shape of dolomite powder was identified as a trigonal system in Figure 2a. The PDF0 specimen’s morphology after 90 days is depicted in Figure 2b. The reference specimen developed some microcracks and micropores. In other words, the PDF0 specimen’s structure was relatively loose. Additionally, a few spherical hollow particles precipitated in the vicinity of the micro-pore. According to the morphology analysis, the spherical hollow particles could be identified as fly ash. After 90 days, the morphology of cement pastes containing 50% dolomite powder was observed in Figure 2c. Cement paste containing dolomite powder exhibited a more compact structure than the PDF0 specimen. Cuboidal hydrate phases (shown in blue circles in Figure 2c) precipitated in the vicinity of the micro-pore. In conjunction with Figure 2a, this implied that the cuboidal hydrate phases should be composed of dolomite powder. Thus, dolomite powder was used to fill micro-cracks and micro-pores in cement paste, resulting in the refinement of the cement paste structure.

### 3.2. Chloride-Penetration Resistance Test

**Figure 3:**
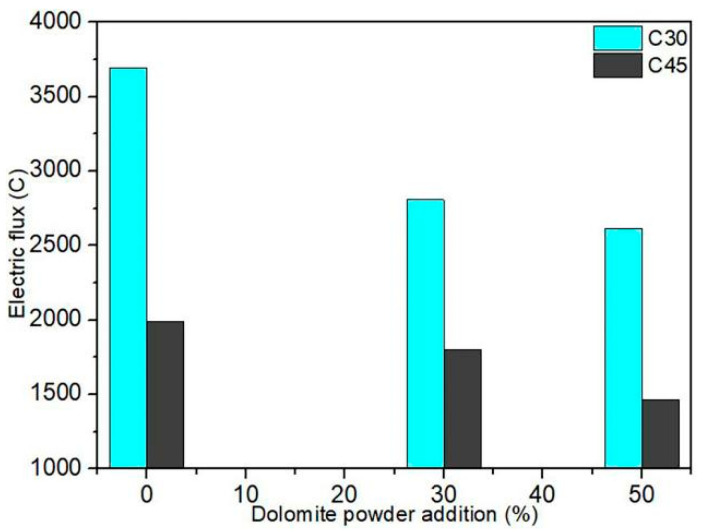
The relationship between the electric flux of concrete and dolomite powder dosage.

Figure 3 depicts the relationship between the electric flux of C30 and C45 concrete and the dosage of dolomite powder. The results indicate that by adding dolomite powder to C30 and C45 concrete, the electric flux was decreased. Electric flux values for CD0-L, CD3-L, and CD5-L specimens of C30 concrete were 3691C, 2808C, and 2615C, respectively. Electric flux values for CD0-H, CD3-H, and CD5-H specimens of C45 concrete were 1991C, 1803C, and 1462C, respectively. It was obvious that resistance to chloride penetration increased. This was because the addition of dolomite powder increased the density of the concrete structure. Thus, by adding dolomite powder, the chloride ion penetration was inhibited, and the impermeability of concrete was increased. This analysis corroborated the SEM test results.

### 3.3. Damage Shapes of Different Freeze–Thaw Cycles

**Figure 4:**
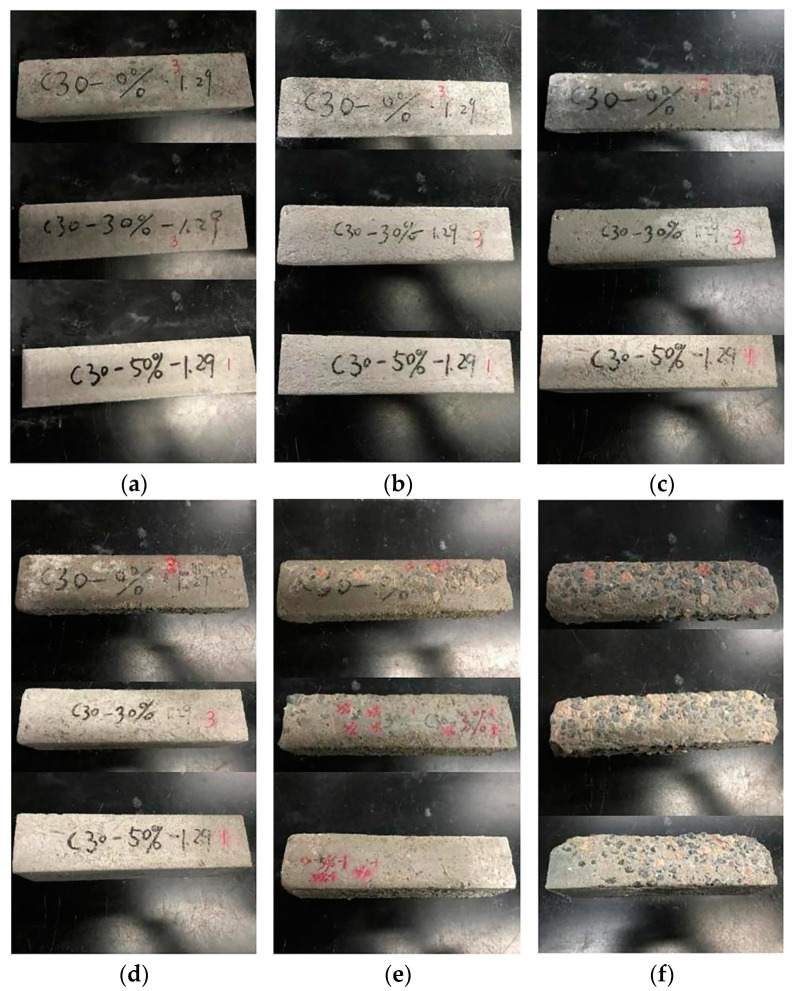
Damage shapes of different freeze–thaw cycles of C30 concretes. (**a**) 0 cycle, (**b**) 50 cycles, (**c**) 150 cycles, (**d**) 200 cycles, (**e**) 225 cycles, (**f**) 300 cycles.

**Figure 5:**
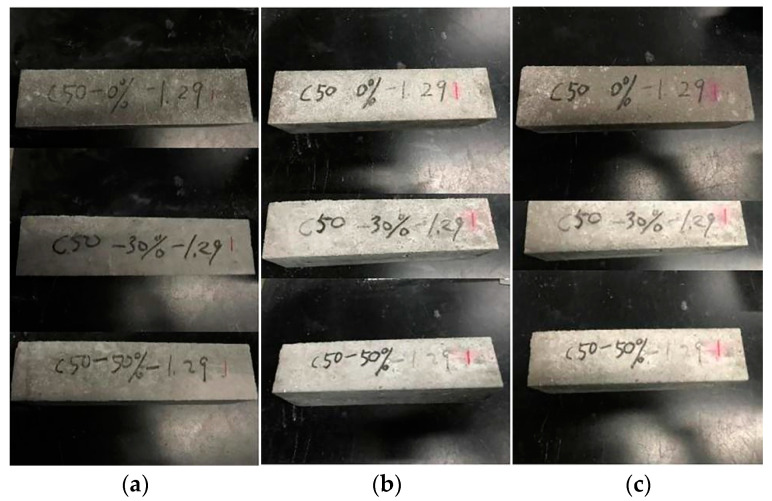
Damage shapes of different freeze–thaw cycles of C45 concretes. (**a**) 0 cycle, (**b**) 250 cycles, (**c**) 300 cycles.

The damage shapes of C30 and C45 concrete with varying compositions at various stages of the freeze–thaw cycle are depicted in Figure 4 and Figure 5. As illustrated in Figure 4, the increased frequency of freeze–thaw cycles accelerated the deterioration of C30 concrete. Concrete had a smooth surface prior to the freeze–thaw cycles. After 50 freeze–thaw cycles, the concrete began to peel away from the surface, and a small pit formed. When the freeze–thaw cycles reached 150, a significant amount of mortar fell off the concrete surface, enlarging the pit area, particularly in the left area of the CD0-L specimen’s surface. After 200 cycles, the coarse aggregate within the reference specimen was gradually exposed to the surface, whereas the coarse aggregate had not yet appeared on the surface of dolomite-powder-infused concrete. When the freeze–thaw cycle was repeated 225 times, a large amount of coarse aggregate was exposed on the reference specimen’s surface, and numerous small pits were connected to form large pits. The CD3-L specimen sustained relatively minor damage compared to the CD0-L specimen, and a small amount of coarse aggregate was exposed on the surface of the CD5-L specimen following 225 cycles. After 300 freeze–thaw cycles, the mortar spalling area spread to the entire surface of the concrete. Meanwhile, the mortar on the concrete surface was nearly imperceptible, and the specimens’ edges had been damaged. In general, increasing the dosage of dolomite powder reduced the degree of damage to concrete caused by freeze–thaw cycles. The surface of C45 concrete was relatively intact and smooth, and the mortar on the concrete surface did not peel off evidently before 250 cycles, as illustrated in Figure 5a–c. After 300 freeze–thaw cycles, a small amount of mortar began to peel away from the reference specimen’s surface, whereas there was no apparent mortar peeling away from the dolomite powder-infused concrete. C45 concrete has a lower damage degree than C30 concrete when subjected to the same freeze–thaw condition.

### 3.4. Mass Loss Rate of Different Freeze–Thaw Cycles

**Figure 6:**
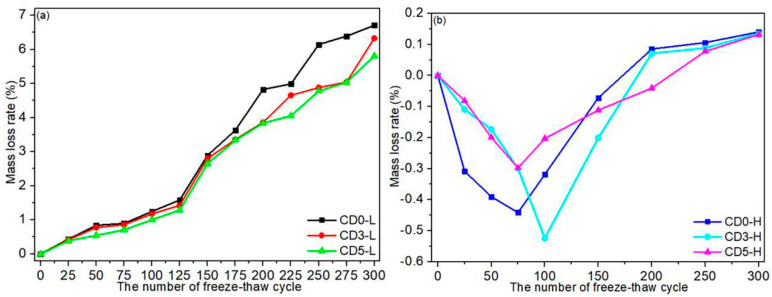
The relationship between the mass loss rate and the number of freeze–thaw cycles of concretes incorporating dolomite powder: (**a**) C30 concretes; (**b**) C45 concretes.

Figure 6 depicts the mass loss rate of concrete due to freeze–thaw cycles. The mass loss rate of C30 concrete is depicted in Figure 6a. As illustrated in Figure 6a, the rate of mass loss from C30 concrete increased as the number of freeze–thaw cycles increased. When the number of freeze–thaw cycles was low, the rate of mass loss was gradual. After 150 freeze–thaw cycles, the mass loss rate of C30 concrete increased significantly. When freeze–thaw cycles were repeated 25 times, the mass loss rates of C30 concrete with various dolomite powder dosages ranged from 0.39% to 0.44%. After 150 cycles, the mass loss rates were determined to be 2.65–2.89%. Meanwhile, the mass loss rates of C30 concrete decreased as dolomite powder dosage was increased. After 25 cycles, the mass loss rates were 0.44%, 0.41%, and 0.39% for CD0-L, CD3-L, and CD5-L specimens, respectively. When the freeze–thaw cycle was repeated 300 times, the mass loss rates of concrete increased to 6.71%, 6.33%, and 5.81% for dolomite powder at 0%, 30%, and 50% replacement levels, respectively. The mass loss rate experiment indicated that the reference specimen of C30 concrete was close to failing after 225 freeze–thaw cycles, whereas concrete blended with dolomite powder failed after 250 freeze–thaw cycles [31]. This result was determined by the damage shapes associated with various freeze–thaw cycles depicted in Figure 4. It demonstrated that dolomite power slowed the mass loss rate of C30 concrete. The mass loss rate of C45 concrete is depicted in Figure 6b. As a result, the development of the concrete’s mass loss rate due to freeze–thaw cycles varied between C30 and C45. Prior to 150 cycles, the mass loss rate grew at a negative rate, but increased to a positive rate after 150 cycles. Due to the low water-to-cement ratio in C45 concrete, the structure was relatively dense. When the number of freeze–thaw cycles was low, C45 concrete suffered minor damage. The concrete contained gel holes, pores, and bubbles. During freeze–thaw cycles, the water and ice in the concrete pores were constantly transformed, resulting in volume expansion [34,35,36]. As a result, the porosity of C45 concrete increased. Water molecules migrated continuously into the concrete’s interior, increasing the moisture content. Thus, when the number of cycles was low, the mass of C45 concrete increased. The freeze–thaw cycles severely damaged the concrete after 150 cycles. Increased concrete mass was insufficient to compensate for the loss. The mass loss rates of CD0-H, CD3-H, and CD5-H specimens were 0.09%, 0.07%, and −0.04%, respectively, when 200 freeze–thaw cycles were performed. After 300 cycles, the mass loss rate of C45 concrete was 0.14%, while concrete incorporating dolomite powder lost mass at a rate of 0.13%. These results demonstrate that the addition of dolomite powder reduced the degree of damage to C45 concrete during freeze–thaw cycles.

### 3.5. Relative Dynamic Elastic Modulus of Different Freeze–Thaw Cycles

**Figure 7:**
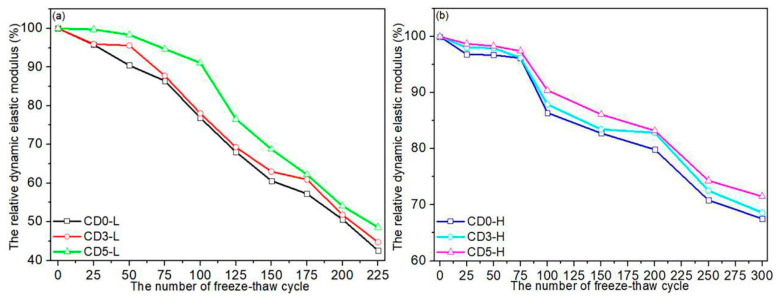
The relationship between the relative dynamic elastic modulus and the number of freeze–thaw cycles of concretes incorporating dolomite powder: (**a**) C30 concretes; (**b**) C45 concretes.

The relationship between the $E_{r}$ and the number of freeze–thaw cycles of the concrete is depicted in Figure 7. The $E_{r}$ is a critical parameter in the study of freeze–thaw cycles because it can be used to characterize the degree of damage within the concrete [37]. In general, the $E_{r}$ of concrete decreased steadily as the number of cycles increased. As illustrated in Figure 7a, the $E_{r}$ of C30 concrete after 50 cycles was approximately 90.45–98.36%. It indicated that the internal damage to C30 concrete was relatively minor after 50 cycles. After 125 cycles, the $E_{r}$ decreased to 68.08–76.58%, indicating that the $E_{r}$ decreased significantly and the concrete’s internal structure was severely damaged. After 225 freeze–thaw cycles, the mortar peeled away from the concrete’s surface, exposing many coarse aggregates. Meanwhile, after 225 cycles, the $E_{r}$ could not be accurately measured. The CD0-L specimen approached the failure standard after 150 cycles, whereas the CD3-L and CD5-L specimens reached the failure standard after 175 cycles.

Combining the results of the mass loss rate test in Figure 6 implied that the mass loss rate test for concrete had a lower sensitivity than the relative dynamic elastic modulus test. When the freeze–thaw cycle was 25, $E_{r}$ values for CD0-L, CD3-L, and CD5-L specimens were 95.79%, 95.95%, and 99.74%, respectively. After 225 cycles, the $E_{r}$ values of CD0-L, CD3-L, and CD5-L specimens reached 42.57%, 44.72%, and 48.56%, respectively. It was discovered that the $E_{r}$ values increased as the dosage of dolomite powder was increased. Figure 7b depicts the relationship between the relative dynamic elastic modulus and the number of freeze–thaw cycles of C45 concrete with various mix proportions. The freeze–thaw damage to C45 concrete was slowed by increasing the dolomite powder dosage. After 25 freeze–thaw cycles, the $E_{r}$ values of CD0-H, CD3-H, and CD5-H specimens were 96.82%, 98.05%, and 98.72%, respectively. The $E_{r}$ values of specimens containing 0%, 30% and 50% dolomite powder were 67.54%, 68.62% and 71.50%, respectively, after 300 cycles. The results of mass loss rate and relative dynamic elastic modulus experiments in Figure 6 and Figure 7 indicate that C45 concrete did not meet the failure standard after 300 freeze–thaw cycles. This phenomenon occurred due to the compact nature of the structure of C45 concrete with a lower water–binder ratio.

### 3.6. The Influence Mechanism of Water-to-Binder Ratio and Dolomite Powder on the Concrete Properties

The volume of the pore solution of concrete increased during freeze–thaw cycles due to the phase change of the bound water caused by the alternate action of positive and negative temperatures. Volume expansion forced the pore solution into unfrozen pores, resulting in the formation of hydrostatic pressure. Hydrostatic pressure annihilated the internal structure of the concrete. Increased freeze–thaw cycles accelerated the growth of cracks and pores, resulting in the superficial degradation of the concrete [38,39]. As illustrated in Figure 4 and Figure 5, the spalling area of mortar spread to the entire surface of C30 concrete after 300 cycles, whereas no visible mortar peeling off occurred with C45 concrete, indicating that the damage degree of C45 concrete was less than that of C30 concrete under the same freeze–thaw condition. It was consistent with the mass loss rate and relative dynamic elastic modulus values obtained from various freeze–thaw cycles. As illustrated in Figure 6, after 300 cycles, the mass loss rates of CD0-L, CD3-L, and CD5-L specimens could increase to 6.71%, 6.33%, and 5.81%, respectively. Meanwhile, the mass loss rates of CD0-H, CD3-H, and CD5-H specimens were 0.14%, 0.13%, and 0.13%, respectively. Figure 7 showed that after 200 cycles, the $E_{r}$ values of CD0-L, CD3-L, and CD5-L specimens reached 50.61%, 51.79%, and 54.09%, respectively. On the other hand, the $E_{r}$ values of CD0-H, CD3-H, and CD5-H specimens were 79.86%, 82.87%, and 83.24%, respectively. In conclusion, the degree of freeze–thaw damage to concrete decreased as the water-to-binder ratio increased. A lower water–binder ratio enhanced mechanical properties and pore refinement, resulting in increased impermeability and decreased water absorption. As a result, there was less freezing water inside the specimen, and the concrete demonstrated increased freeze–thaw resistance [37,40,41]. As illustrated in Figure 4 and Figure 5, the degree of damage to C30 and C45 concrete blended with dolomite powder was relatively low in comparison to reference specimens. The SEM and chloride-penetration resistance tests revealed that the addition of dolomite powder refined the pore structure of concrete and increased its impermeability. Additionally, the concrete containing dolomite powder peeled slightly away from the surface during freeze–thaw cycles, as the formation of pores and cracks in concrete was inhibited. Thus, the degree of damage caused by hydrostatic pressure on concrete blended with dolomite powder was limited during freeze–thaw cycles. This analysis corroborated the mass loss rate and relative dynamic elastic modulus results. Dolomite powder slowed the mass loss rate of C30 and C45 concrete, while increasing the $E_{r}$ of the concrete. All of these findings indicate that increasing the dosage of dolomite powder improved the freeze–thaw resistance of concrete.

### 3.7. Damage Model of Concrete Incorporating Dolomite Powder Based on Weibull Distribution

**Figure 8:**
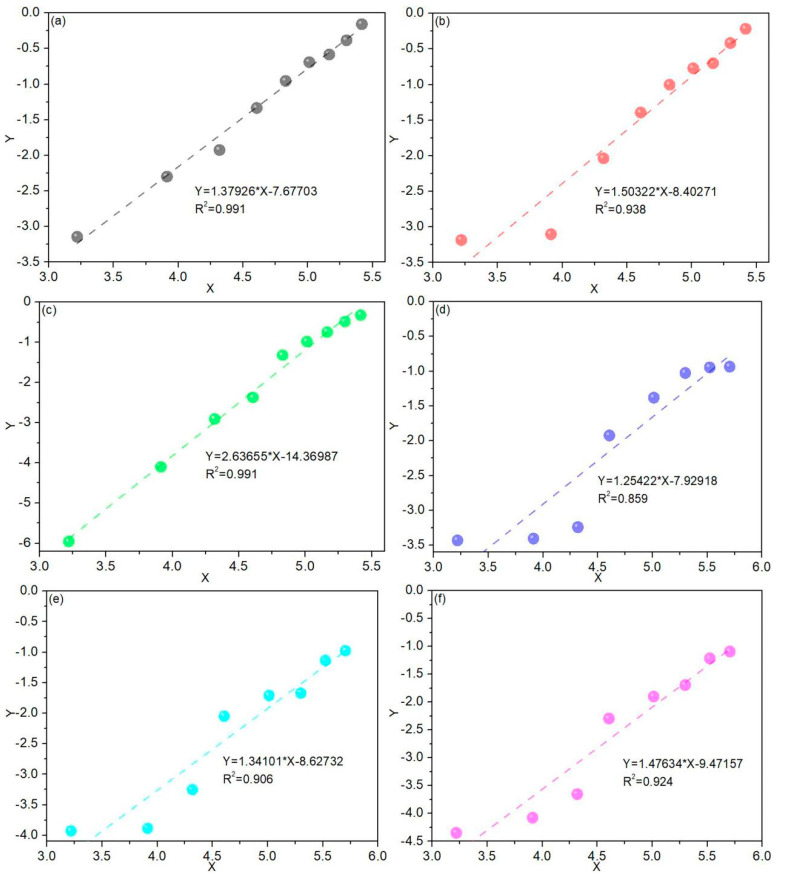
Graphical analysis of the freeze–thaw damage for: (**a**) CD0-L; (**b**) CD3-L; (**c**) CD5-L; (**d**) CD0-H; (**e**) CD3-H; (**f**) CD5-H.

**Figure 9:**
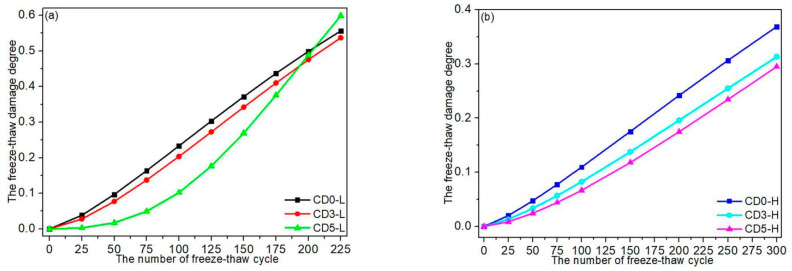
Evolution model of the damage degree of concretes for various replacement levels of dolomite powder: (**a**) C30 concretes; (**b**) C45 concretes.

Figure 8 depicts a graphical analysis of concrete’s freeze–thaw damage. The parameters of Equation (9) were determined using linear regression analysis between $\mathrm{In}\left(\mathrm{In}\frac{1}{1-D_{N}}\right)$ and InN. Table 6 summarizes the Weibull distribution’s characteristic parameters. As illustrated in Figure 8 and Table 6, the damage degree of concrete blended with dolomite powder was found to follow the two-parameter Weibull probability function during freeze–thaw cycles. As illustrated in Figure 9, an evolution model of concrete damage for various dolomite powder replacement levels was developed using Table 6 and Equation (6). As a result, the curves of C30 and C45 concrete with dolomite powder were generally lower than those of the reference concrete. Under the same freeze–thaw conditions, the damage degree of concrete treated with dolomite powder was less than that of reference specimens. It demonstrated that increasing the dosage of dolomite powder resulted in increased freeze–thaw resistance. Equations (10)–(15) show the evolution model formula for the damage degree of CD0-L, CD3-L, CD5-L, CD0-H, CD3-H, and CD5-H specimens, respectively.

(10) $$D_{N}=1-exp[-\left(\frac{N}{261.39951}{)}^{1.37926}\right]$$

(11) $$D_{N}=1-exp[-\left(\frac{N}{261.39951}{)}^{1.37926}\right]$$

(12) $$D_{N}=1-exp[-\left(\frac{N}{232.81754}{)}^{2.63655}\right]$$

(13) $$D_{N}=1-exp[-\left(\frac{N}{556.68577}{)}^{1.25422}\right]$$

(14) $$D_{N}=1-exp[-\left(\frac{N}{622.31688}{)}^{1.34101}\right]$$

(15) $$D_{N}=1-exp[-\left(\frac{N}{611.29215}{)}^{1.47634}\right]$$

According to material science, the damage degree of concrete was reduced to 40% after several freeze–thaw cycles, which could be considered a material failure [42]. Table 7 shows the number of rapid freeze–thaw concrete cycles with material failure when the $D_{N}$ of concrete is 40% based on Equations (10)–(15). As shown in Table 7, the number of rapid freeze–thaw cycles performed on CD0-L, CD3-L, and CD5-L specimens with material failure was 161, 171, and 180, respectively. Meanwhile, the number of rapid freeze–thaw cycles performed on CD0-L, CD3-L, and CD5-L specimens with material failure was 150, 175 and 175, respectively. The calculated value for C30 concrete was induced to be close to the experimental value. As a result, a damage model for concrete incorporating dolomite powder based on the Weibull distribution could accurately reflect the degree of damage sustained by the concrete during rapid freeze–thaw cycles. The number of rapid freeze–thaw cycles performed on CD0-H, CD3-H, and CD5-H specimens with material failure was 326, 377 and 389, respectively, according to the Weibull distribution. The service life of C30 and C45 concrete can be calculated using the average annual number of actual field freeze–thaw cycles, the number of rapid freeze–thaw cycles, and their equivalent relationship [43]. As a result, the service life of concrete incorporating dolomite powder was sufficient to be evaluated using a damage model based on the Weibull distribution (as seen in Equations (10)–(15)).

## 4. Conclusions

In this work, the freeze–thaw resistance and damage model of C30 and C45 concretes with different dolomite powder dosages were investigated. The following conclusions could be drawn:

Under the freeze–thaw cycles, the damage degree of C30 and C45 concretes blended with dolomite powder was restrained due to the refinement of the cement paste structure with the addition of dolomite powder. The evolution model of the damage degree based on the two-parameter Weibull probability function showed that the damage degree of concretes decreased with the addition of dolomite powder under the same freeze–thaw conditions.

The evolution model of the damage degree of concretes was proposed, which was expressed in terms of the number of the freeze–thaw cycles. The comparison between experimental and calculated values indicated that the evolution model of the damage degree of concretes based on the two-parameter Weibull probability function could be used to forecast and evaluate the service life of concretes blended with dolomite powder. This manuscript provides a new path to improve the service life of concretes from the perspective of freeze–thaw resistance for readers. Therefore, researchers can improve the service life by designing an evolution model of the damage degree of concretes based on the two-parameter Weibull probability function in future work. It is worth noting that deep studies on slight differences in service life between experimental and calculated values have rarely been reported up to now. It is necessary to further optimize the evolution model of the damage degree of concretes based on the two-parameter Weibull probability function at a later stage, especially for building materials with high durability requirements.

## Figures and Tables

**Table 1:** Chemical composition of Portland cement (wt.%).

LOI	SiO_2_	Al_2_O_3_	CaO	f-CaO	Fe_2_O_3_	MgO	Na_2_O	SO_3_	Cl
1.33	22.37	4.36	61.08	0.86	3.38	2.43	0.51	2.45	0.01

**Table 2:** Chemical composition of dolomite powder (wt.%).

CO_2_	N	Na_2_O	MgO	Al_2_O_3_	SiO_2_	SO_3_	K_2_O	CaO	Fe_2_O_3_	SrO
45.97	1.08	0.02	18.62	0.21	0.03	0.04	0.02	33.65	0.04	0.02

**Table 3:** Chemical composition of fly ash (wt.%).

TiO_2_	K_2_O	CeO_2_	MgO	Al_2_O_3_	SiO_2_	SO_3_	P_2_O_5_	CaO	Fe_2_O_3_	SrO
1.08	1.00	0.09	1.87	30.72	51.20	0.78	0.59	5.53	4.70	0.09

**Table 4:** Mix proportions of cement pastes (wt.%).

Specimen	w/b	Dolomite	Cement	Fly Ash	Water
PDF0	0.36	0	1	0.25	0.36
PDF3	0.36	0.375	1	0.25	0.36
PDF5	0.36	0.625	1	0.25	0.36

**Table 5:** Mix proportions of concretes-the amount of ingredients in concrete of per cubic meter.

Specimen	w/b	Dolomite (kg/m^3^)	Cement (kg/m^3^)	Fly ash (kg/m^3^)	Water (kg/m^3^)	Sand (kg/m^3^)	Gravel (kg/m^3^)	Superplasticizer (kg/m^3^)
CD0-L	0.55	0	276	69	190	640	1188	0
CD3-L	0.55	97	256	64	176.2	597	1188	0
CD5-L	0.55	155	246	61	169	573	1188	0
CD0-H	0.36	0	350	88	157.7	640	1188	17
CD3-H	0.36	120	324	81	145.8	600	1188	20
CD5-H	0.36	190	307	78	138.6	560	1188	21

**Table 6:** Weibull distribution characteristic parameters.

Sample	β	a	α	R^2^
CD0-L	1.37926	–7.67703	261.39951	0.991
CD3-L	1.50322	–8.40271	267.68401	0.938
CD5-L	2.63655	–14.36987	232.81754	0.991
CD0-H	1.25422	–7.92918	556.68577	0.859
CD3-H	1.34101	–8.62732	622.31688	0.906
CD5-H	1.47634	–9.47157	611.29215	0.924

**Table 7:** The number of rapid freeze-thaw cycles of concrete with material failure.

Sample	Calculated Value	Experimental Value
CDF0-L	161	150
CDF3-L	171	175
CDF5-L	180	175
CDF0-H	326	-
CDF3-H	377	-
CDF5-H	389	-

## Data Availability

Data available in a publicly accessible repository. The data presented in this study are openly available in [https://wwt.lanzouj.com/iAq9E00hl8li].
